# The Moral Foundations of Child Health and Social Policies: A Critical Interpretive Synthesis

**DOI:** 10.3390/children8010043

**Published:** 2021-01-13

**Authors:** Avram E. Denburg, Mita Giacomini, Wendy J. Ungar, Julia Abelson

**Affiliations:** 1 Department of Paediatrics, The Hospital for Sick Children, Toronto, ON M5G 1X8, Canada; 2 Peter Gilgan Centre for Research and Learning, The Hospital for Sick Children, Toronto, ON M5G 1X8, Canada; wendy.ungar@sickkids.ca; 3 Institute of Health Policy, Management and Evaluation, University of Toronto, Toronto, ON M5T 3M6, Canada; 4 Centre for Health Economics and Policy Analysis, Department of Health Research Methods, McMaster University, Hamilton, ON L8S 4L8, Canada; giacomin@mcmaster.ca (M.G.); abelsonj@mcmaster.ca (J.A.)

**Keywords:** child, health policy, public policy, social values

## Abstract

Background: Allusions to the uniqueness and value of childhood abound in academic, lay, and policy discourse. However, little clarity exists on the values that guide child health and social policy-making. We review extant academic literature on the normative dimensions of child health and social policy to provide foundations for the development of child-focused public policies. Methods: We conducted a critical interpretive synthesis of academic literature on the normative dimensions of child health and social policy-making. We employed a social constructivist lens to interpret emergent themes. Political theory on the social construction of target populations served as a bridge between sociologies of childhood and public policy analysis. Results: Our database searches returned 14,658 unique articles; full text review yielded 72 relevant articles. Purposive sampling of relevant literature complemented our electronic searches, adding 51 original articles, for a total of 123 articles. Our analysis of the literature reveals three central themes: potential, rights, and *risk*. These themes retain relevance in diverse policy domains. A core set of foundational concepts also cuts across disciplines: well-being, participation, and best interests of the child inform debate on the moral and legal dimensions of a gamut of child social policies. Finally, a meta-theme of embedding encompasses the pervasive issue of a child’s place, in the family and in society, which is at the heart of much social theory and applied analysis on children and childhood. Conclusions: Foundational understanding of the moral language and dominant policy frames applied to children can enrich analyses of social policies for children. Most societies paint children as potent, vulnerable, entitled, and embedded. It is the admixture of these elements in particular policy spheres, across distinct places and times, that often determines the form of a given policy and societal reactions to it. Subsequent work in this area will need to detail the degree and impact of variance in the values mix attached to children across sociocultural contexts and investigate tensions between what are and what ought to be the values that guide social policy development for children.

## 1. Background

Most societies attach special importance to children and childhood. Allusions to the uniqueness and value of childhood abound in academic, lay, and political discourse. Yet social policies affecting children across a range of domains often fail to reflect the priority ascribed to children [1,2,3]. In the field of health care, policies on drug development, regulation, and funding routinely neglect issues unique to children, in advanced and developing systems alike [4,5]. In the realm of education, mounting emphasis on adult economic attainment in early childhood policies arguably constrains pedagogies centered on caring relationships and geared toward meeting diverse childhood needs [6]. Despite progressive changes in sociocultural perceptions of children with disability in most advanced societies, much of childhood disability policy and programming remains premised on narrowly delimited medical criteria for access [7]. Child welfare policies can induce greater focus on the protection of societal norms about ideal family structures and environments than on the protection of children themselves [8,9]. Social spending in a broad cross-section of advanced nations tilts heavily toward older populations and away from children [2,10]. These diverse examples disclose a tension between tacit social values and explicit policy that warrants investigation.

A nuanced understanding of the moral foundations of child-focused policies is an important first step in this endeavor. Little clarity exists in popular, political, or scholarly spheres on the values that guide child health and social policymaking. How do our societies value health gains in children? When does a child’s autonomy outweigh the interests of his family members? How and in what ways should children participate in policy and program decisions that affect them? The answers to these and allied questions rest on the foundational values that shape how we portray and involve children in our societies. In this paper, we review extant academic literature on the normative dimensions of child health and social policy to provide evidence-informed foundations for the development and adjudication of child-focused health and social policies.

## 2. Methods

We conducted a qualitative systematic review of academic literature on the normative dimensions of health and social policy-making for children. The search strategy followed a critical interpretive synthesis (CIS) approach, which balances the rigor and sensitivity to the quality of systematic review methodologies with the capacity for depth and breadth of inductive qualitative approaches [11,12]. A “compass” question guided our search of the literature: What ethical and social values inform health and social policies for children? (Figure S1). Based on concepts contained in our primary search question, we developed a matrix of Boolean-linked keywords and iteratively refined an optimal search strategy with guidance from a university librarian with expertise in electronic database search techniques (Table S1). Between March and June of 2018, we searched the following databases, refining search strategies for each platform to optimize yield: OVID Medline, PsychInfo, EMBASE, Web of Science, CINAHL, ProQuest, and LexisNexis (Table S2). We developed a priori inclusion and exclusion criteria to guide article selection based on iterative discussions between study team members. Papers were excluded if they did not provide insight into the normative or values dimensions of social policy for children, either with respect to the content of child health and social policies or the processes by which such policies are made.

To complement the systematic selection of relevant papers from online databases, we employed an inductive qualitative approach to data collection. Purposive sampling of academic literature was conducted in an iterative fashion during the data analysis phase to fill emergent conceptual gaps. This sampling stage was informed by the pre-existing content knowledge of study team members and colleagues, which was supplemented by snowball searches of reference lists from key publications. This dual approach to the selection and refinement of relevant literature facilitated the reflexivity and ongoing interpretive synthesis at the heart of the CIS approach [13].

Data analysis proceeded through four phases. First, we identified and categorically coded the major concepts and values in each included article. Second, using constant comparative methods, we worked interpretively across conceptual and normative categories to develop “synthetic constructs” that rendered each category in light of the whole body of evidence surveyed [14]. Third, we sought to attend to points of tension and discordance within and among the constructs and to consider their meaning. Finally, we built a synthesizing argument based on the insights from the interaction of these constructs, out of which theoretical insights emerged. We employed a social constructivist lens to frame, juxtapose, and interpret themes emerging from the diffuse literature on public policy for children [15,16]. Political theory on the social construction of target populations served as a bridge between sociologies of childhood and public policy analysis (Table S3) [17,18]. Our analysis of child health and social policies proceeded from the contention that the social construction of children as a target population—one often framed by vulnerability—has a strong influence on policy agendas and design, and inversely, that such policies embed constructed messages about children that influence society’s perceptions of them and the social issues at hand.

## 3. Results

Our database searches returned 14,658 unique articles, of which 342 met inclusion criteria upon review of titles and abstracts. Full-text review yielded 72 relevant articles. A purposive sampling of relevant literature complemented our electronic searches and was refined in light of the emergent results from data analysis, adding an additional 51 original articles, for a total of 123 articles (Figure S2).

**Figure 1:**
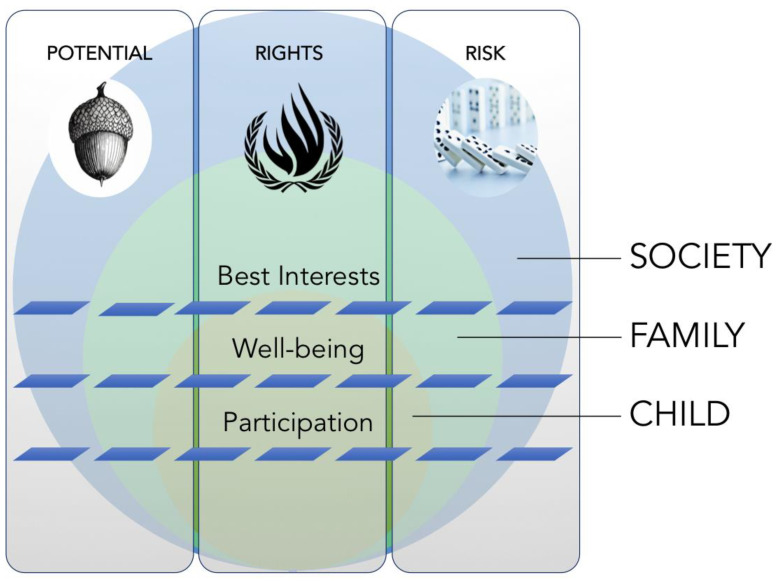
The moral scaffolding of child health and social policy.

Our review of the literature exposes few explicit analyses of the normative foundations of child health and social policy. Formal attempts to name, interrogate, or prioritize select values—either generally or in specific policy domains—are rare. Three central themes, each encompassing a few key values, emerge from the literature: potential, rights, and risk. The theme of potential captures discourse on childhood as a developmental state angled toward adulthood and the evolving capacity implied by this trajectory. Rights relate to ideas, normative and legal, about the human rights held by children, which have gained prominence over recent decades. Risk incorporates ideas about vulnerability and the corollary need for protection that animate scholarship about children and childhood across a range of disciplines. A set of established foundational concepts related to social policy for children—well-being, participation, and best interests of the child—cuts across and links these central themes. We distinguish these concepts from the themes above to emphasize their use as fundamental tropes within and across the various studies in the literature that explore ideas related to childhood potential, rights, and risk. They constitute the ideational goals toward which scholarship on social policy for children recurrently angles; whereas, the themes themselves surface the dynamic tensions inherent in pursuit of those goals. Finally, an overarching theme of embedding—both familial and societal—emerges from the academic discourse in all policy domains examined, which provides structural context for the expression and resolution of these tensions. It gives form to the pervasive issue of a child’s place, in the family and in society, at the heart of much social theory and applied analysis on children and childhood (Figure 1).

### 3.1. Potential

The idea of latent or unrealized potential inherent in children dominates in much of the literature. Allusions to childhood as a “state of becoming” cross disciplinary bounds and policy domains, as do justifications for policy agenda setting, development, and implementation premised on the realization of childhood potential. A number of distinct, if overlapping, sub-themes surface recurrently. Notions of futurity and arguments for investment in children are inherent in both theoretical discourse about childhood and applied analyses of a range of child-centered policies [19]. These arguments emphasize and often explicitly value children’s potential to contribute to society as eventual adults—especially as “return on investment” in economic terms—and leverage this idea as guidance for policy formulation [20,21]. Critically, this future orientation often eclipses valuing the present needs, experiences, and perspectives of children [22,23]. Such constructs closely align with the core values and assumptions of economic liberalism, wherein productive work and economic contribution epitomize social capital. Related to this are frequent equations of childhood with preparation. Childhood is routinely construed as a preparatory stage of life that framed as both an opportunity and a means to socialize the young into prevailing societal norms and expectations [24].

Varied policy domains invoke the idea of the child’s potential in different ways. Both “return on investment” and health promotion have served as key normative frames for child health policy debate. Evidence suggests that these tropes have helped disaggregate children from other disadvantaged groups and produce the political consensus necessary to move policy initiatives on health coverage for children forward [25]. Childhood potential has also been invoked in health policy discourse to address national security concerns. Successive child health coverage expansions in the US relied heavily on policy frames such as early vulnerability, human potential, future economic contribution, and, in particular, long-term national economic productivity and military strength [26]. Notions of return on investment also prevail in the scientific and policy literature on early childhood development early childhood development. Mounting knowledge about the impacts of early childhood environments and experiences on brain development and long-range cognitive outcomes has underwritten the development of policy arguments based on future potential and ultimate economic contribution [27]. By contrast, in the field of child welfare, the available evidence on policy impact at the population level is comparatively thin, so discourse focuses on extrapolating moral arguments from individual cases to broader child welfare policies. Moralistic frames predominate: arguments based on desert, rather than outcomes, have often held sway [28,29].

### 3.2. Rights

Rights-based language figures prominently in the academic literature concerned with the moral dimensions of public policy for children. Rights have the broadest disciplinary reach, mirroring the 20th century ascendance of human rights legislation and jurisprudence in national and international spheres of governance. Much of the literature draws on discourse and tenets from the United Nations Convention on the Rights of the Child (CRC), which is the principal child rights covenant of modernity [30]. The ratification of the CRC dramatically increased the volume and changed the tenor of academic scholarship on children’s rights. The construct of “the competent child” has emerged, which is an image focused on the child as a rights-bearing individual: one with legitimate needs and preferences, the right to voice them, and the right to participate in decisions about how to meet them. Notably, while the discourse on “potential” focuses on the effects of policy, rights discourse introduces issues of policy process; the participatory rights of children and the inclusion of their voice in policy decisions impacting them are fundamental concerns. This discourse strains traditional notions—common in the child protection movement and couched in the rhetoric of risk—of the child as a passive, incomplete, and ultimately incompetent vessel in need of protection and edification [31].

The literature reveals important synergies between child rights and two paradigmatic normative concerns attached to policy formulation and adjudication for children: well-being and best interests. A parochial definition of well-being conceives it as the absence of abuse, neglect, exploitation; a more expansive definition focuses minimally on need and optimally on inclusive, holistic definitions of a high quality of life [32,33]. Conceptions of child well-being in academic discourse have evolved from narrowly conceived ideas related to the protection of the most vulnerable in the 19th and early 20th centuries to expansive ideas about well-being couched in the universal rights of children [34]. The justification for child well-being has evolved in tandem from one founded in charity to one premised on entitlement. The broad acceptance achieved by the CRC has tied notions of child well-being to achievement of their social, cultural, economic, civil, and political rights [35], and it has tempered culturally relativistic renderings of children’s purpose and well-being [36].

A parallel narrative centered on participation emerges in the literature, which sets in relief the role of rights in changing ideas about children’s well-being and best interests. Changing mores about children, founded in changing models of the young child, have influenced ideas about the legitimacy and necessity of involving children in policy decisions that affect them. Child rights are one of the principal drivers of changing societal perceptions [37]. Relatedly, recent insights in the field of child development studies have contributed to major changes in conceptual models of the young child, with corresponding implications for, and impacts on, ideas about involving children in policy decision-making. Scholars have identified three dominant models of the young child—the child as possession, the child as subject, and the child as qualified participant—and have elaborated a new model of the child as social actor, which is founded in novel theory and evidence from a diverse array of disciplines [38]. CRC principles and jurisprudence buttress this model: United Nations General Comment No. 7 elaborates an explicit accounting of a child’s right to expression in “the development of policies and services, including through research and consultations” [39]. The upshot has been a progressive incorporation of ideas of autonomy and participation into ideas about children’s well-being and best interests: in policy domains as diverse as predictive genetic testing, sexuality and sexual health, child welfare, public health, and research involving children; and in forms as varied as a seat at the policy table, proxy communication through identified advocates, and the incorporation of research evidence on children in policymaking.

Even so, a number of tensions are inherent in the relationship between conceptions of children’s rights and their best interests. The interface of child and parental rights remains a murky ethical and legal zone. The values of child autonomy and participation can conflict with the legitimacy of parental discretion in decisions regarding children in the child’s best interests. Child rights scholars offer a hierarchical taxonomy of intergenerational rights in response, with parental rights as derivative from child rights and therefore “functional” in nature [40]. This formulation recasts parental rights as prerogatives in the service of responsibilities insofar as they protect and advance the child’s rights [41].

Allied issues relate to the substance and application of a child’s right to participate. What are the best ways to enact children’s participation in policy development? What does participation look like in practical terms? The literature reveals divergent views about the intent and form of legitimate child participation, with identified problems ranging from tokenism, to degrees of imbalance in power relations, to issues of equity in opportunities for expression. Critics note that “rights-thinking” abstracted from social context induces myopia on structural barriers to rights execution. Some argue that the practical instantiation of rights implies degrees of autonomous capacity that many children lack due to sociopolitical constraints, such as poverty, ethnic or cultural marginalization, familial mores, or lack of political franchise. In Huntington’s words, “The dominant conception of rights is one-sided in its emphasis on individualism, rather than relationships” [42].

Corrective attention to social embedding comes through most clearly and consistently in the public health literature. Scholarship on public health policy invokes twin imaginings of children as rights-bearing individuals and relational beings, with attendant tensions between the two [43]. One view affirms (evolving) moral agency, the other recognizes the embedded and contingent nature of childhood within family and societal institutions: an exclusive focus on rights can divorce public health policies for children from engagement with the lived realities of childhood, with corollary implications for equity and impact [44]. An instructive example issues from the realm of childhood obesity policy. Some scholars prescribe programs with the intent for universal reach, such as public education campaigns, in deference to the ubiquity of the problem. Others contend that programs, which emphasize health education above specific policy levers, such as food taxes, will tend to marginalize families and communities with less baseline capacity to act on educational prescriptions, such as low-income and rural groups [45].

Relatedly, health care policy literature addressing difficult ethical issues about the value of life tests the limits of child rights in relation to their family and social context. Newborn and infant rights are a case in point. Inquiry into cultural intuitions about the value of newborn life—studied through institutional policies and stakeholder perceptions attached to neonatal intensive care—reveals (and problematizes) an instance in which beliefs seem to shift from a defense of rights as unassailable entitlements to socially contingent ones [46]. Categorical distinction of the value of newborn life from other child life underscores the moral contingency attached to child health. Vague notions of “personhood” are leveraged to weigh the merits of acute medical intervention (e.g., resuscitation) for the neonate [47]. Corollary considerations about the burdens imposed on other family members by newborn needs are incorporated into judgments about distributive justice within families in respect of both parents and siblings. Whether an acutely ill infant should live or die often rests on the results of such arithmetic. Such patterns of policy thought and clinical practice expose deeply embedded historical, evolutionary, and sociocultural factors that ground societal perceptions about the value of newborn and infant rights to life. Scholarly documentation of these and other instances of the relational character of child rights open critical windows into the landscape in which our social values about children move. Despite contentions, the direction of movement is clear: rights language has woven itself intimately into the fabric of academic and political discourse about public policy for children, and it is certain to texture policy formulation and implementation into the future.

### 3.3. Risk

Risk is a central theme linking social values to policies for children, its expression varying by discipline and domain of social policy [48]. Innocence is a frequent precursor to notions of risk. Representations of the child as primitive and innocent abound in popular, scholarly, and political cultures, with either positive and utopian or negative (feral, delinquent) connotations [1]. Innocence relates closely to notions of vulnerability and protection, as well as to the conception of childhood as a preparatory period of “socialization” discussed above. Allusions to vulnerability shape a common view of childhood as inherently risky. Vulnerability discourse is marked in the childhood development and welfare literatures. Insights from developmental science identify sensitive periods during which early experiences can have an outsized influence on developmental trajectories, especially cognitive, psychological, and physiological patterns of behavior [49,50]. The child-as-vulnerable also prefigures but draws inspiration from theories and evidence on maternal–child bonding in developmental psychology [51]. Permanency is a closely related idea that has predominated in child welfare discourse and policy-making. Child welfare scholars and advocates theorize that stability in early childhood environments allows for bonding with a “psychological parent” that diminishes risk in early childhood and fosters improved developmental outcomes [52]. The confluence of these perceived determinants of risk—innocence, vulnerability, and a need for relationship permanency—induces an emphasis on protection and provision as natural domains for social policy touching on children. Protection from abuse and neglect has served as a hegemonic principle in social work and child welfare systems across disparate polities for much of the past century [48].

Themes about protection from risk relate closely to the concepts of children’s well-being and best interests. The discourse linking these concepts to child protection issues from both the child welfare and public health fields, with varying definitions. As noted above, well-being receives both narrow and expansive formulation. Its negative notion conceives well-being as freedom from abuse, neglect, and exploitation. In positive terms, it is measured by response to need and aimed at maximizing quality of life [32]. Critics have argued that the almost singular emphasis on a narrative of protection from risk in child welfare has excluded broader notions of child well-being, which are attentive to structural determinants of health and human flourishing [29]. They emphasize the socially and historically contingent nature of well-being: one tied to family functioning and parental responsibility, influenced by human rights paradigms, and variably constrained by protection of the private sphere [33]. From this standpoint, the risk/protection nexus constrains the ambit of what child well-being could represent and how policy should seek to realize it.

The relationship between protection and best interests is more intimate still. This connection emerges most clearly from the child health and welfare fields, in which the concept of “best interests of the child” (BIC) has served as a dominant moral and legal yardstick [53]. The standard assimilates concepts related to protection from harm and promotion of welfare, and it centers on an assessment of the balance between the benefits and risks of an intervention or policy [54]. The health care field has long adjudicated clinical or research interventions in children by reference to BIC. A recurring theme in the academic discourse on best interests centers on their legitimate scope: achieved, by turns, through the juxtaposition of individual, family, and population perspectives. In the realm of research involving children, a tension emerges between the protection of children, as a uniquely vulnerable population, and the promotion of aggregate child welfare through advancements in scientific knowledge [55]. Lags in child-centered health research—particularly in the realm of drug and technology development—have challenged the definition of BIC as protection of the individual child from research-related harm, widening its scope to encompass the harms suffered by populations of children from constraints on scientific progress [56].

BIC is also a central standard in the ethics of clinical practice. However, again, the locus of interests accounted for—those of child, parent, or family—complicates the interpretation of BIC. A prime example relates to genetic testing in children [57]. When genetic disease is not amenable to prevention or mitigation during childhood proper, the BIC standard has often dictated the deferral of such testing until such time as the child can make an informed decision about it [58,59,60]. Questions surrounding the handling of incidental results from whole genome sequencing, and the rights of family members to knowledge of such results, have challenged the traditional understanding of best interests [61]. A tension is evident between notions of family-embeddedness and the evolving autonomy and capacity of children [62,63]. On the one hand, the ascendance of rights paradigms has induced a conflict between paternalism and participation in the interpretation of a best interests standard: some understand fidelity to best interests as fulfillment of the totality of CRC-enshrined rights, with due emphasis placed on autonomy [62]. Others see the interests of a child as “embedded in and dependent on the interests of the family unit” and argue for the incorporation of parental and family interests in that standard [58]. To wit, the benefits that accrue to family members from the disclosure of incidental results about genetic disease in asymptomatic minors enter into the moral calculus governing the handling of genetic knowledge and weigh against corollary risks to the child.

Debate on the legitimate bounds of a best interests standard also turn on different conceptions of risk. Again, the ramifications of genetic testing provide a useful case study. Arguments to withhold incidental findings about genetic conditions center on worries about alterations to parent–child relationships: the risk of sundered bonding from changed perceptions about one’s child outweigh potential benefits from such knowledge [63]. Conversely, those who argue for the disclosure of incidental results justify their position through reference to the medical risks of undetected conditions. Relatedly, scholars and practitioners have defended the decision to grant parental requests for predictive genetic testing of their children through allusion to familial psychosocial risks related to uncertainty about a child’s genetic inheritance. The prevention or resolution of “disabling parental anxiety” counts in the tally of a child’s best interests [61].

Child welfare scholarship and case law have also routinely employed BIC as a means to measure the need for, and justify interventions to enhance, child protection. The concept itself has roots in English feudal law and relates to the doctrine of parens patriae: the king as father of his people. Initially employed to legitimate sovereign wardship over “natural fools and idiots”, it was gradually expanded to include state duty toward the protection of children [54]. The best interests standard has come to serve, in most liberal democracies, as a bulwark against historically unfettered parental possessory rights. A child’s best interests have become an elemental facet of legal decisions—and popular sensibility—regarding the protection and well-being of children in society. The institutionalization of rights discourse, culminating in the CRC, has underwritten this tendency: the rights of the child imply specific corollary duties—of the parent, of society—that justify the curtailment of certain freedoms [64].

Signal debates in the child welfare literature issue from the intermingled interests of children, the rights and duties of parents, and the role of the state. Some scholars detect myopia in the hegemonic interpretation of best interests as “child protection” in social work and child welfare systems [65]. Protection from parental abuse and neglect dominates the prescribed hierarchy of child interests and leaves little room for more inclusive notions of well-being that are attentive to the social determination of health [66]. As Walsh notes: “Focus on child abuse and the subsequent construction of ‘child protection’… has contributed to the creation of ‘neglected oppressions’ of age, illness, disability and poverty in the acceptance of those who are seen to be ‘in need’” [29]. In parallel, there is a foundational struggle between participation and paternalism in child welfare services: a complex dynamic exists where the state’s responsibility to safeguard children from harm meets its duty to promote their participatory rights. This tension turns on the intrinsic vulnerability assigned to children, and the consequent pull between competing views of the child as “the powerless victim of the malice of adults” and “the potential unlocker of solutions” [37]. In the wake of a child’s rights revolution, social theorists have begun to detail portraits of children as active social agents rather than passive recipients of circumstance, and to argue for social policy that empowers them to enact this agency [34].

A survey of risk discourse across this broad range of disciplines and subjects yields a landscape of childhood marked by its vulnerability and populated by attempts to build in norms of protection. Protection from harm—in the home environment, in institutional contexts such as health care, human subjects research, and law, in broader economic and political systems—is frequently justified, and judged, by reference to ideas about children’s well-being and best interests. As with potential and rights, risk is relational: it is situated in family and societal contexts and calibrated against the interests of each.

## 4. Discussion

Our review exhibits the prominence of three core themes—potential, rights, and risk—and established concepts—well-being, best interests, and participation—across diverse academic disciplines and policy areas (Figure 1). The boundaries of these themes are at times indistinct: ideas about potential, rights, and risk move across disciplines and interact within them, alternately reinforcing and challenging one another (Table S4). Their relationship with well-being, best interests, and participation is also variable. Scholars invoke notions of well-being, best interests, and participation more explicitly in discourse on child rights and risk than in relation to arguments about childhood potential. However, they are not absent in the latter. Implicit ideas about well-being and best interests proceed from teleological views of the child: well-being is equated with optimal development into adulthood, and policies are seen to align with a child’s best interests insofar as they promote this end. Notably, the academic literature has tended to examine best interests and well-being in isolation from one another; their interaction is little explored. A view from above renders them as overlapping—though not transposable—concepts that derive from distinct historical and institutional trajectories: well-being largely from public health, and best interests from legal traditions in child welfare and human subjects research. The potential to draw on conceptual synergies between the two is abundant but largely untapped.

The embedded nature of childhood is a foundational and unifying theme across diverse disciplines and subjects. Childhood is conditioned by layered structures of family, community, and society; images of and debates about children are framed by recognition of this contingent state. In particular, conceptions of well-being and best interests of the child are routinely tied to the well-being and interests of the family and, in certain instances, to broader societal well-being. Dominant ideas about childhood potential, couched in terms of future social and economic contribution, blur the boundaries between individual and societal well-being in policy frames used to justify interventions in early childhood education and child health. The framing and adjudication of risk in childhood—for instance, as evinced in policy debates on pediatric genetic testing and child welfare—are closely allied to ideas about parent–child roles and responsibilities to one another, and how these impact relational interests within families. Child rights discourse grapples with the foundational tension between the sanctity and contingency of personhood, as capacities evolve and the contours of personhood solidify. This tension is evident in the use of rights arguments in debates on a wide range of child health and social policy domains, including research involving children, genetic testing, and public health interventions.

### 4.1. Policy Neglect

Much of the literature reviewed, irrespective of policy domain, describes situations of relative neglect with respect to robust public policy for children. Our analysis suggests that while the reasons for such neglect vary somewhat by political and cultural context, reliable features emerge, chief among them institutional and ideational factors. Consistent institutional constraints that surface across polities and policy worlds are the absence of political voice for children and the comparative lack of strong institutions designed to protect and advocate for children [23,25,37]. Ideational challenges to public policy advances for children are often located in states of competition with other minority groups for political attention [17]. On a number of social policy issues, children are absorbed into negative social constructions that frame other groups (notably, the poor and minorities) to which their parents belong [2]. The splintering of children into multiple sub-populations may dilute the effect of the positive societal associations attached to children per se. Despite recognition of the policy neglect attached to children, insights into the need for more sophisticated policy arguments are rare in the existing literature.

### 4.2. Rhetorical Shifts

Our review also identifies shifts across time and place in the rhetoric used to justify public policy for children. These shifts hint at the influence of historical and cultural context on the expression and impact of values on policy in a given jurisdiction or domain. For instance, US child policy rhetoric has shifted from arguments drawing on notions of rights, obligations, and compassion to economic arguments that leverage cost/benefit calculus [67]. This rhetorical shift has both political and sociocultural roots, including declining religiosity, the rising hegemony of empirical evidence in policymaking, and fluctuating fiscal pressures [68]. Evidence suggests there has been a gradual overall rise in the use of economic reasoning to frame and justify child policy, and a corollary decrease in moralistic reasoning [2]. The speed and size of such changes vary by policy domain: as discussed above, moralistic arguments initially predominated in policy on education and poverty but gradually gave way to economic arguments; by contrast, US policymakers have long framed child health policies in utilitarian logic and couched their value in economic terms.

Modern welfare states are at various points along a discursive trajectory from welfare to well-being as an ordering principle for child-centered social policy. In most, visions of the child as “weak, poor, and needy” have historically underwritten policy prescriptions; in a few, such visions give way to more holistic conceptions of well-being [1]. In Britain, policies governing children’s services in a range of domains emphasize well-being as an objective, variably incorporating notions of need, deprivation, rights, quality of life, and social standing in its definition and measurement [65]. Scotland’s recent development of a national policy framework for children places well-being at its center [35]. The social–democratic universalism long at work in most Scandinavian countries has tended to induce a focus on need fulfillment, rather than risk mitigation, in social policy: for children and families, this has meant inclusive and positive definitions of child well-being at the core of social policies for children [69,70].

Although rhetoric differs in tone and emphasis across jurisdictions, there are broad trends evident; our analysis captures the most prominent and impactful of these. The increasing reliance on future potential, often expressed in economic language, is evident across the majority of liberal democracies. Mounting allusion to child rights and well-being as yardsticks of successful social policy is also broadly detectable, and it has gained global traction in the form of international child rights covenants and broadly adopted social indicators [71,72]. However, the relative concentration of literature on policies and populations in the Global North precludes a truly global perspective on the relationship between values and child policies. The vast majority of the world’s children live in political and cultural contexts where the impacts of child social policies have received little rigorous attention, scholarly or otherwise. Cross-cultural contestations of the values that ground public policy for children, and the consequent means and ends of such policies, are still poorly understood [73].

### 4.3. Limitations

Our analysis of the values dimensions of social policy for children is limited by language in two ways. Firstly, we restricted our searches and analysis to English language literature. As a result, we may have captured a limited proportion of the existing cultural variance in values construction and emphasis vis-à-vis children and childhood. Secondly, the broad bounds of this work meant dealing with very different disciplinary languages. The play of each theme discussed varies by policy domain, depending on the tropes and accrued debates of the field. Abstracting from the specific context of such debate limits appreciation of the varied ways in which key issues are conceived and addressed.

Finally, our insights are constrained somewhat by the limited number of studies that explore the views and values of children themselves. In the few studies that directly involve or report on child and youth perspectives, emphasis on the a priori value of childhood experience predominates. As compared to adult counterparts, youth participants tend to accord less attention and import to instrumental justifications for child and family policies—for instance, child care as a means to adult economic productivity, or education as a means to subsequent economic contribution—and more to policies responsive to the intrinsic value of childhood [74]. When solicited, their policy preferences emphasize increasing child and youth “voices” in policy discourse, re-conceptualizing education as a means to more robust citizenship, environmental protections, and policies and programs to empower youth [75]. The lack of literature that gives voice to child and youth perspectives on the values and goals that should shape public policies with direct bearing on them is a critical gap in many fields of scholarship on children. We hope our findings help motivate efforts to fill this gap.

## 5. Conclusions

The insights that emerge from the broad themes we identify suggest more coherence than fragmentation in the normative concerns attached to children and childhood. We—as academics, as policymakers, as citizens of a collective—recursively frame and adjudicate policies for children in the light of a narrow band of moral presuppositions. Most societies paint children as potent, vulnerable, entitled, and embedded. It is the admixture of these elements in particular policy spheres, across distinct places and times, that determines the form of a given policy and societal reactions to it. Absent an understanding of these core values, our capacity to learn from past policy failures and project future successes is fundamentally crippled.

Foundational understanding of the moral language and dominant policy frames applied to children can enrich future analyses of existing and proposed social policies for children in a range of sociopolitical contexts. Potential applications are readily apparent. Better understanding of the ways in which societies value health gains in children—does their intrinsic value outweigh instrumental considerations? Are gains made now less valuable if they fail to promote long-term potential?—could help set system priorities for funding health technologies and services. More nuanced evidence on modes and perceptions of child participation in social policy agenda-setting and development could inform context-specific criteria for engaging children and youth in political decisions that affect them. Subsequent work in this area will need to detail the degree and impact of variance in the values mix attached to children across sociocultural contexts.

## Data Availability

All data generated or analysed during this study are included in this published article and its Supplementary Materials files.

## Supplementary Materials

The following are available online at https://www.mdpi.com/2227-9067/8/1/43/s1, Figure S1: Research questions, Figure S2: Literature sampling process and yield, Table S1: Child policy values CIS search strategy, Table S2: Sample search strategy: MEDLINE, Table S3: Social construction of target populations, Table S4: Relationship between policy domain and values. All data generated or analysed during this study are included in this published article and its supplementary information files.

## References

[1] Moss P., Petrie P. (2002). From Children’s Services to Children’s Spaces: Public Policy, Children and Childhood.

[2] Gormley W. (2012). Voices for Children: Rhetoric and Public Policy.

[3] Brown A.D., Katherine W., Allen K., Quach U., Chiu E., Bialystok L. (2010). Turning the social determinants of health to our advantage: Policy fundamentals for a better approach to children’s health. Healthcare Q..

[4] Denburg A.E., Ungar W.J., Greenberg M. (2017). Public drug policy for children in Canada. Can. Med. Assoc. J..

[5] Denburg A., Arora B., Arora R.S., Auste C., Bagai P., Barr R., Challinor J., Eden T., Grynzspancholc E., Hoffman R. (2017). Access to essential medicines for children with cancer: A joint SIOP-CCI position statement. Lancet Oncol..

[6] Wild M., Silberfeld C., Nightingale B. (2015). More? Great? Childcare? A discourse analysis of two recent social policy documents relating to the care and education of young children in England. Int. J. Early Years Educ..

[7] Halfon N., Houtrow A., Larson K., Newacheck P.W. (2012). The changing landscape of disability in childhood. Future Child..

[8] Mass M., van Nijnatten C. (2005). Child protection and the conception of parental responsibility. Am. J. Orthopsychiatr..

[9] Onheiber M. (1997). Toward a reorientation of values and practice in child welfare. Child. Psychiatr. Hum. Dev..

[10] Lynch J. (2006). Age in the Welfare State: The Origins of Social Spending on Pensioners, Workers, and Children.

[11] Dixon-Woods M., Cavers D., Agarwal S., Annandale E., Arthur A., Harvey J., Hsu R., Katbamna S., Olsen R., Smith L. (2006). Conducting a critical interpretive synthesis of the literature on access to healthcare by vulnerable groups. BMC Med. Res. Methodol..

[12] Boyko J.A., Lavis J.N., Abelson J., Dobbins M., Carter N. (2012). Deliberative dialogues as a mechanism for knowledge translation and exchange in health systems decision-making. Soc. Sci. Med..

[13] Moat K., Lavis J.N., Abelson J. (2013). How contexts and issues influence the use of policy-relevant research syntheses: A critical interpretive synthesis. Milbank Q..

[14] Glaser B.G., Strauss A.L. (1967). The Discovery of Grounded Theory: Strategies for Qualitative Research.

[15] Berger P., Luckmann T. (1966). The Social Construction of Reality.

[16] Holstein J.A., Gubrium J.F. (2007). Constructionist Perspectives on the Life Course. Sociol. Compass.

[17] Schneider A., Ingram H. (1993). The social construction of target populations. Am. Political Sci. Rev..

[18] Ingram H., Schneider A., deLeon P., Sabatier P.A. (2007). Social Construction and Policy Design. Theories of the Policy Process.

[19] Knijn T., van Oorschot W. (2008). The need for and the societal legitimacy of social investments in children and their families: Critical reflections on the Dutch case. J. Fam. Issues.

[20] Kahn A., Kamerman S.B., Phipps S., Ben-Arieh A. (2009). From Child-Saving to Child Development. From Child Welfare to Child Well-Being: An International Perspective on Knowledge in the Service of Policy-Making.

[21] Waldfogel J. (2006). What do children need?. Public Policy Res..

[22] Klaus A. (1993). Every Child a Lion: The Origins of Maternal and Infant Health Policy in the United States and France, 1890–1920.

[23] Mayall B. (2006). Values and assumptions underpinning policy for children and young people in England. Child. Geogr..

[24] Hatch J.A. (1995). Studying Children as a Cultural Invention: A Rationale and a Framework. Qualitative Research in Early Childhood Settings.

[25] Sardell A. (1990). Child Health Policy in the U.S.: The Paradox of Consensus. J. Health Politics Policy Law.

[26] Rosenbaum S., Mauery D.R., Shin P., Hidalgo J. (2005). National Security and US Child Health Policy: The Origins and Continuing Role of Medicaid and EPSDT.

[27] Millei Z., Jones A. (2014). The Australian Early Childhood Curriculum and a Cosmopolitan Imaginary. Int. J. Early Child..

[28] Nicholson R.H. (1994). What is a child worth?. Häst. Cent. Rep..

[29] Walsh T. (1999). Changing expectations: The impact of ‘child protection’ on Irish social work. Child. Fam. Soc. Work.

[30] Convention on the Rights of the Child (CRC) G.A. Res. 44/25 (1989). http://www2.ohchr.org/english/law/crc.htm.

[31] Cannella G.S., Swadener B.B. (2006). Contemporary public policy influencing children and families: “Compassionate” social provision or the regulation of others?. Int. J. Educ. Policy Res. Pr. Reconcept. Child. Stud..

[32] Ager A. (2013). Annual research review: Resilience and child wellbeing public policy implications. J. Child. Psychol. Psychiatr..

[33] Seaberg J.R. (1990). Child well-being: A feasible concept?. Soc. Work.

[34] Reading R., Bissell S., Goldhagen J., Harwin J., Masson J., Moynihan S., Parton N., Santos Pais M., Thoburn J., Webb E. (2009). Promotion of children’s rights and prevention of child maltreatment. Lancet.

[35] Coles E., Cheyne H., Rankin J., Daniel B. (2016). Getting It Right for Every Child: A National Policy Framework to Promote Children’s Well-being in Scotland, United Kingdom. Milbank Q..

[36] Doek J., Ben-Arieh A., Casas F., Frones I., Korbin J. (2014). Child Well-Being: Children’s Rights Perspective. Handbook of Child Well-Being: Theories, Methods and Policies in Global Perspective.

[37] Sanders R., Mace S. (2006). Agency policy and the participation of children and young people in the child protection process. Child. Abus. Rev..

[38] MacNaughton G., Hughes P., Smith K. (2007). Young Children’s Rights and Public Policy: Practices and Possibilities for Citizenship in the Early Years. Child. Soc..

[39] OHCHR General Comment No. 7 (2005: 01/11/2005). Implementing Child Rights in Early Childhood. http://www.ohchr.org/english/bodies/crc/comments.htm.

[40] Reynaert D., Bouverne-de-Bie M., Vandevelde S. (2009). A Review of Children’s Rights Literature Since the Adoption of the United Nations Convention on the Rights of the Child. Child. Glob. J. Child. Res..

[41] Howe R.B. (2001). Do parents have fundamental rights?. J. Can. Stud..

[42] Huntington C. (2006). Rights myopia in child welfare. UCLA Law Rev..

[43] Purcell M. (2010). Raising healthy children: Moral and political responsibility for childhood obesity. J. Public Health Policy.

[44] Kolhatkar G., Berkowitz C. (2014). Cultural considerations and child maltreatment. In search of universal principles. Pediatr. Clin. N. Am..

[45] Branka S., Nikogosian H., Lobstein T. (2007). The Challenge of Obesity in the WHO European Region and the Strategies for Response.

[46] Janvier A., Bauer K.L., Lantos J.D. (2007). Are Newborns Morally Different from Older Children?. Theor. Med. Bioeth..

[47] Burkhart J. (1989). The social construction of Personhood. Soc. Thought.

[48] Jenson J., Fraser M. (2006). Social Policy for Children and Families: A Risk and Resilience Perspective.

[49] Halfon N., Hochstein M. (2002). Life course health development: An integrated framework for developing health, policy, and research. Milbank Q..

[50] Huston A.C. (2005). Connecting the science of child development to public policy. Soc. Policy Rep. Soc. Res. Child. Dev..

[51] Graham H. (1980). Prevention and Health: Every Mother’s Business’. A comment on child health policies in the 1970s. Sociol. Rev..

[52] Sribnick E.G. (2011). The origins of modern child welfare: Liberalism, interest groups, and the transformation of public policy in the 1970s. J. Policy Hist..

[53] Kopelman L.M. (1997). The best interests standard as threshold, ideal, and standard of reasonableness. J. Med. Philos..

[54] Shah S. (2013). Does research with children violate the best interests standard? An empirical and conceptual analysis. Northwest. J. Law Soc. Policy.

[55] Tauer C.A. (2002). Central ethical dilemmas in research involving children. Account. Res..

[56] Ross L.F. (2008). Ethical and policy issues in pediatric genetics. Am. J. Med. Genet. Part. C Semin. Med. Genet..

[57] Zlotnik S.R., Ungar W.J. (2016). Maximizing the benefit and mitigating the risks of moral hazard. Am. J. Bioethics.

[58] Ross L.F., Saal H.M., David K.L., Anderson R.R., American Academy of and the American Academy of Pediatrics, American College of Medical Genetics and Genomics (2013). Technical report: Ethical and policy issues in genetic testing and screening of children. Genet. Med..

[59] ESHG (2009). Genetic testing in asymptomatic minors: Recommendations of the European Society of Human Genetics. Eur. J. Hum. Genet..

[60] Borry P., Stultiens L., Nys H., Cassiman J.J., Dierickx K. (2006). Presymptomatic and predictive genetic testing in minors: A systematic review of guidelines and position papers. Clin. Genet..

[61] Clayton E.W., McCullough L.B., Biesecker L.G., Joffe S., Friedman Ross L., Wolf S.M. (2014). Addressing the ethical challenges in genetic testing and sequencing of children. Am. J. Bioethics.

[62] Zawati M.H., Parry D., Knoppers B.M. (2014). The best interests of the child and the return of results in genetic research: International comparative perspectives. BMC Med. Ethics..

[63] Hardart G.E., Chung W.K. (2014). Genetic testing of children for diseases that have onset in adulthood: The limits of family interests. Pediatrics.

[64] Vandergrift K., Bennett S. (2012). Children’s rights: A framework for health promotion. Healthc. Q..

[65] Axford N. (2009). Child well-being through different lenses: Why concept matters. Child. Fam. Soc. Work.

[66] Raphael D. (2010). The health of Canada’s children: Public policies and the social determination of children’s health. Paediatr. Child. Health.

[67] Sardell A. (2014). Insuring Children’s Health: Contentious Politics and Public Policy.

[68] Yarrow A.L. (2011). A history of federal child antipoverty and health policy in the United States since 1900. Child. Dev. Perspect..

[69] Thevenon O. (2011). Family policies in OECD countries: A comparative analysis. Popul. Dev. Rev..

[70] Lundberg O., Yngwe M.Å., Stjärne M.K., Elstad J.I., Ferrarini T., Kangas O.E., Norström T., Palme J., Fritzell J. (2008). The role of welfare state principles and generosity in social policy programmes for public health: An international comparative study. Lancet.

[71] Ben-Arieh A., Ferran C., Frones I., Korbin J.E., Ben-Arieh A., Casas F., Frones I., Korbin J. (2014). Multifaceted Concept of Child Well-Being. Handbook of Child. Well-Being: Theories, Methods and Policies in Global Perspective.

[72] Kamerman S.B., Phipps S., Ben-Arieh A. (2009). From Child Welfare to Child Well-Being: An International Perspective on Knowledge in the Service of Policy-Making.

[73] LeVine R.A., New R.S., LeVine R.A., New R.S. (2008). Introduction. Anthropology and Child Development. A Cross-Cultural Reader.

[74] Piker A. (2011). Balancing liberation and protection: A moderate approach to adolescent health care decision-making. Bioethics.

[75] Michalski J.H. (1999). Values and Preferences for the ‘Best Policy Mix’ for Canadian Children.

