# Supplementary material for: The Moral Foundations of Child Health and Social Policies: A Critical Interpretive Synthesis
Source: Children (Basel). 2021 Jan 13;8(1):43. doi: 10.3390/children8010043 (PMC7828333; doi:10.3390/children8010043)
Supplement: Supplementary file 1 [file children-08-00043-s001.pdf]

Supplementary

Research Questions

Descriptive:

- What values undergird health and social policymaking for children?
- Does the policy domain influence the use and framing of values regarding children? Do certain values predominate in specific policy domains?

Interpretive:

- Where do scholarly accounts of values in child social policies overlap and diverge? What points of consonance and tension are evident?
- How do scholars juxtapose and reconcile discrete values?

Search question:

- What ethical and social values inform health and social policies for children?

Figure S1. Research questions.

Table S1. Child policy values CIS search strategy.

Synonyms for social values	AND	Synonyms for policy	AND	Context domain synonyms	AND	Synonyms for child
**Value*** (to capture: value, values)		**Policy, policies, policymaking** (to capture: policy, policies, policymaking, policy-making)		**Public*** (to capture: public, publics, publicly, public policy)		**Child*** (to capture: child, children, childhood, child care, childcare)
**Ethic*** (to capture: ethic, ethics, ethical)				**Social*** (to capture: social, social policy, social policies)		**P*ed*** (to capture: pediatric, pediatrics, paediatric, paediatrics)
**Norm*** (to capture: norm, norms, normative)				**Health*** (to capture: health, health care, health-care, health care, health system, health-system, health systems, health sector, health care sector, health-care sector, health care sector)		**Infant*** (to capture: infant, infants)
**Preference*** (to capture: preference, preferences)				**Welfar*** (to capture: welfare, welfarist, welfare policy)		**Adolescen*** (to capture: adolescent, adolescents, adolescence)
**Priorit***				**Educat***		**Teen***

(to capture: priority, priorities, prioritization)	(to capture: educate, educating, education, educational, education policy)	(to capture: teen, teenage, teen-age, teenager, teen-ager)
	**Development*** (to capture: development, developmental, early childhood development)	
	**Right*** (to capture: right, rights, human rights, child rights)	
	**Econom*** (to capture: economic, economics, econometric, economy, economies, economic policy)	

Table S2. Sample search strategy: MEDLINE.

**Ovid MEDLINE(R) 1946 to Present with Daily Update**			
**#**	**Searches**	**Results**	**Search Type**
1	Social Values/	18692	Advanced
2	Social Norms/	138	Advanced
3	exp Ethics/	131609	Advanced
4	(value* or norm or norms or normative or ethic* or moral*).tw,kf.	1398111	Advanced
5	1 or 2 or 3 or 4	1481056	Advanced
6	exp Public Policy/	119129	Advanced
7	Policy Making/	13240	Advanced
8	(policy* or policies).tw,kf.	156581	Advanced
9	6 or 7 or 8	238720	Advanced
10	exp infant/ or (infant* or infancy or newborn*or new-born* or baby* or babies or neonat*).tw,kf. or exp child/ or (child* or kid or kids or toddler*).tw,kf. or exp adolescent/ or (adoles* or teen* or boy* or girl*).tw,kf. or minors/ or exp pediatrics/ or (paediatric* or pediatric*).tw,kf.	3421475	Advanced

11	5 and 9 and 10	6186	Ad-vanced

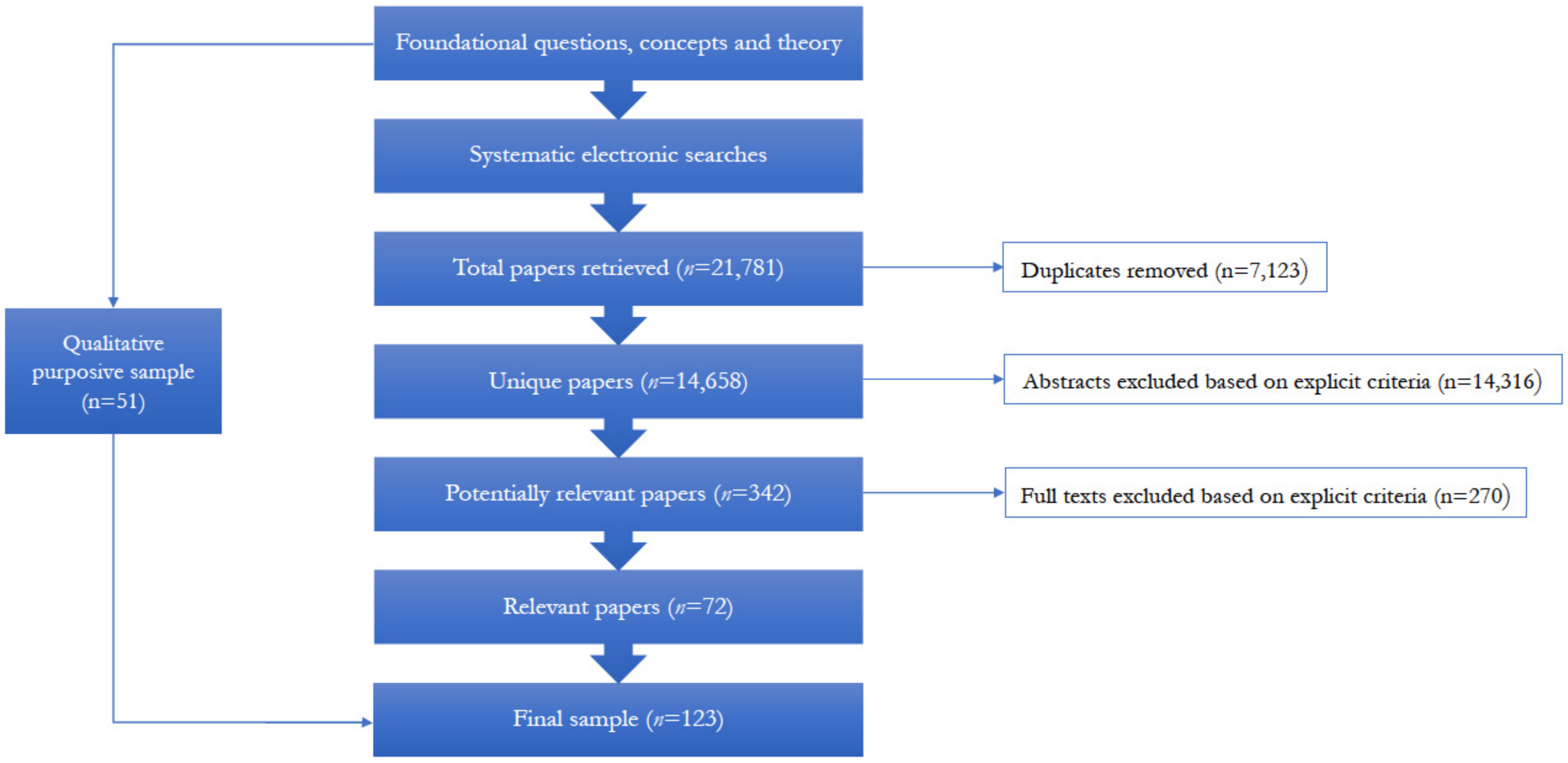

Figure S2. Literature sampling process and yield.

Table S3. Social construction of target populations.

		CONSTRUCTIONS	
		**Positive**	**Negative**
POWER	**Strong**	*Advantaged*	*Contenders*
		Elderly<br>Business<br>Scientists	Rich<br>Minorities<br>Cultural elites
	**Weak**	*Dependents*	*Deviants*
		Children<br>Mothers<br>Disabled	Criminals<br>Drug addicts<br>Gangs

Adapted from Schneider and Ingram 1993.

Table S4. Relationship between policy domain and values.

Policy domain	Dominant values/themes	Points of tension	Illustrative quotes
**Genetic testing**	• Best interests of the child<br>• Risk/benefit ratio<br>• Relational obligations, family embedding<br>• Child rights: autonomy, capacity, participation	• Pull between notions of family-embeddedness and evolving autonomy and capacity of children<br>• Tension between loci of interests accounted for: child vs parent/family	• "The interest of a child is embedded in and dependent on the interests of the family unit."[1] (p.238)<br>• "One important consequence of explicitly incorporating family interests into the best interest calculation is that an already difficult and subjective determination of the limits of parental authority will unavoidably become messier and more difficult."[2] (p.238)
		• Conflict between paternalism and participation in the interpretation of best interests standard	• "The best interests of the child framework is increasingly complicated by a growing appreciation of pediatric autonomy and the development thereof."[3] (p.72)
		• Competing risks: altered parent-child relationship through knowledge of genetic mutation vs. psychosocial risk to family of uncertainty around genetic knowledge vs. medical risk of undetected genetic condition	• "It may be ethically acceptable to proceed with predictive genetic testing to resolve disabling parental anxiety…"[15] (p.238)<br>• "…any risk of altered parental nurturing as a result of receiving information is outweighed by the increased ability of the child to recognize the need to obtain medical care in the future."[4] (p.5)
**Child welfare**	• Well-being: negative and positive formulations<br>*Negative*: absence of abuse, neglect, exploitation<br>*Positive*: minimally, focused on need; maximally, inclusive of expansive definitions of quality of life and desire<br>• Protection<br>• Agency and participation: tethered to child rights principles/conventions	• Multifaceted and internally tense notion of child well-being, incorporating: need, rights, quality of life, material condition, and social embeddedness	• *Need vs quality of life*: need "focused on minimum requirements to avoid harm and participate in society", whereas quality of life "captures degrees of enrichment [and] children's subjective perception of their situation."[5] (p.380)
		• Socially and historically contingent conceptions of well-being: tied to family functioning, influenced by human rights paradigms, and constrained by protection of private sphere of affairs	• *Quality of life vs. rights*: "quality of life offers an important counterbalance to rights, which…can give rise to a plethora of standards and procedures aimed at averting risk."[19] (p.380)
		• 'Child protection' vis-à-vis abuse/neglect as hegemonic principle in social work and child welfare systems, to the exclusion of broader notions of well-being	• "Focus on child abuse and the subsequent construction of 'child protection'…has contributed to the creation of 'neglected oppressions' of age, illness, disability and poverty…in the acceptance of those who are seen to be 'in need'."[6] (p.36)

	• Possessory rights of parents vs. best interests of the child	• Complex dynamic between child rights and parental rights, and between the responsibility of the state to safeguard children from harm and to promote their participatory rights	• "The extremely heavy emphasis in our society on individual freedom of action combines with the historical stress on parental rights in a formidable alliance resisting children's rights."[7] (p.157-8)<br><br>• "The tension within child protection between the child as the powerless victim of the malice of adults and the child as the potential unlocker of the solutions to their own difficulties…"[8] (p.90)
**Health policy (Public health, health care)**	• Distinctness<br><br>• Vulnerability, innocence and protection<br><br>• Potential (life-course), harm prevention<br><br>• Risk/threat to future population health patterns<br><br>• Intrinsic vs. instrumental value of child life<br><br>• Family embeddedness, 'intrafamilial distributive justice'<br><br>• 'Fair innings'<br><br>• Evidence<br><br>• Investment<br><br>• Rights vs. relations<br><br>• Liberalism vs. protectionism in capacity for medical decision-making<br><br>• 'Personhood'	• Internal pull between justifications for prioritization of child health interventions based on intrinsic (distinctness) and instrumental (potential) valuations of child health<br><br>• 'Fair innings' argument privileges children but reduces them to a vessel for aggregate potential: it does not value childhood states of health or well-being differently than those of adults, nor is there a specific accounting made of differentials in developmental impacts across the life-course	• "Childhood as a distinct developmental phase of life…"[9] (p.70)<br><br>• "Society may value health gains in children more highly because of their vulnerability."[10] (p.417)<br><br>• "The child as a site of investment for the future"; "children as 'becomings' and not as 'beings' with experiences in the present."[11] (p.292)
		• Competing conceptions of children as rights-bearing individuals and relational beings	• "Child health is not complete without considering spillover effects and non-health benefits, including changes in parent/caregiver productivity and earnings, family member quality of life and functioning."[24] (p.418)
		• Novel equity implications regarding allocation of scarce health care resources to children vis-à-vis quality and availability of evidence on child health technologies and services to guide allocation decisions	• Priority setting frameworks/methods "may penalize children if the quality of evidence is poorer or scanter than in adults."[24] (p.417)
		• Categorical distinction of the value of newborn life, with historical, evolutionary and sociocultural dimensions	• "The value of a baby's life is determined, in part, by the family context into which he or she is born…The commonness of infant death [has] led to protective cultural and emotional mechanisms in the form of philosophic differentiation of the newborn from older people."[12] (p.418-9)
**Child care, early**	• Teleological justification for child care policies: emphasis on educational outcomes and employability	• Sociopolitical context crucial in interpreting child care and educational policies, as political ideology influences cast and weight of values grounding policy formulation	• Prevailing justifications for policy on early childhood education "produce powerful notions of childhood by emphasizing its futurity and connection with the nation state."[13] (p.67)

**childhood education**	• Instrumental valuation of child care as mechanism for both enhanced child development and promotion of parental/family self-sufficiency (e.g. through employment)	• Varying degrees of ambivalence and/or discord across polities regarding the appropriate role of government in mediating family life and decision-making vis-à-vis children	• "Americans' strongly held values – including the importance of family, work, and equal opportunity…have come to fix the boundaries of public support for government interventions on behalf of very young children."[14] (p.54)
		• Competing justificatory frameworks for early childhood education and care: prevailing neoliberal model of economic potential vs. models emphasizing cosmopolitan belonging and solidarity	• Care and education for children has been more marketised and commodified, with an emphasis on education outcomes rather than relational processes."[15] (p.243)<br>• Alternative frames for early childhood education involve "imagery of the 'social' understanding of other cultures and ways of being, relationships and interdependence between people, and a disposition of openness…"[27] (p.73)
**Research involving children**	• (Minimal) risk<br>• Protection from harm<br>• Equity<br>• Evidentiary demands for promotion of child welfare<br>• Individual vs population harms/protections	• Fundamental tension between protection of children from harm, and promotion of child welfare through advancements in scientific knowledge relevant to children<br>• Differing interpretations about what constitutes acceptable risk to individuals, both as individuals and in service of the collective	• "The welfare of all children depends on research to test the safety and effectiveness of medical procedures, drug and biologics, and public health measures. Such research is essential in order to provide benefits and to prevent harms within the population of children as a whole."[16] (p.128)<br>• "With this protection came a drawback: the health issues unique to children were underfunded and understudied."[17] (p.529)
**Child rights**	• Autonomy, individuality<br>• Participation<br>• Dignity<br>• Parental/family rights<br>• Teleological vs. intrinsic worth	• Increasing focus on image of 'the competent child', as opposed to child as passive, incomplete and ultimately incompetent vessel in need of protection and edification	• The "image of the competent child…is considered as a reaction against the childhood image of the incompetent child, characterized by considering children as objects in need of protection because of their vulnerability."[18] (p.520-1)
		• Tension between child autonomy and participation and legitimacy of parental discretion in decisions regarding children<br>• Divergent views about the intent and form of legitimate child participation	• "The centre stage of the children's rights paradigm is the recognition of the child as an autonomous subject, meaningful in its current 'child-being'…Parental prerogatives derive from the rights of their children."[32] (p.525)
		• Criticisms of 'rights-thinking' as divorced from social context, and therefore naive about structural barriers to rights execution	• "The dominant conception of rights is one-sided in its emphasis on individualism, rather than relationships."[19] (p.664)

List of Articles in Final Review Sample

1. Ager A. Annual research review: Resilience and child wellbeing public policy implications. *The Journal of Child Psychology and Psychiatry* 2013; 54(4): 488-500.
2. Axford, N. (2009). "Child well-being through different lenses: Why concept matters." Child & Family Social Work 14(3): 372-383.
3. Baird, B. (2008). "Child Politics, Feminist Analyses." Australian Feminist Studies 23(57): 291-305.
4. Battle, S. F. and J. R. Battle (1987). "Adolescent sexuality: Cultural and legal implications." Child & Youth Services 9(1): 125-136.
5. Ben-Arieh, A., F. Casas, I. Frones and J. E. Korbin (2014). Handbook of child well-being: Theories, methods and policies in global perspective (Vols. 1-5). Handbook of child well-being: Theories, methods and policies in global perspective (Vols. New York, NY, Springer Science + Business Media; US: 1-5). xxxv, 3258.
6. Ben-Arieh, A. and M. Khoury-Kassabri (2008). "Attitudes toward and understanding of children's rights among middle school students in Jerusalem: the role of family values and patterns, nationality, and religion." American Journal of Orthopsychiatry 78(3): 359-368.
7. Ben-Arieh, A., M. Khoury-Kassabri and M. M. Haj-Yahia (2006). "Generational, ethnic, and national differences in attitudes toward the rights of children in Israel and Palestine." American Journal of Orthopsychiatry 76(3): 381-388.
8. Blacksher, E. (2008). "Children's health inequalities: ethical and political challenges to seeking social justice." Hastings Center Report 38(4): 28-35.
9. Blake, V., S. Joffe and E. Kodish (2011). "Harmonization of ethics policies in pediatric research." Journal of Law, Medicine & Ethics 39(1): 70-78.
10. Borry P, Stultiens L, Nys H, Cassiman JJ, Dierickx K. Presymptomatic and predictive genetic testing in minors: a systematic review of guidelines and position papers. *Clinical genetics* 2006; 70(5): 374–381.
11. Branka S, Nikogosian H, Lobstein T. *The Challenge of Obesity in the WHO European Region and the Strategies for Response*. Copenhagen, Denmark: WHO, 2007.
12. Britto, P. R., N. Ulkuer, W. P. Hodges and M. F. McCarthy (2013). Global policy landscape and early childhood development. Handbook of early childhood development research and its impact on global policy. New York, NY, Oxford University Press; US: 65-81.
13. Brown AD, Katherine W, Allen K, et al (2010). Turning the social determinants of health to our advantage: Policy fundamentals for a better approach to children's health. *Healthcare Quarterly*; 14: 68-75.
14. Burkhart J. The social construction of personhood. *Social Thought* 1989; 15(2): 21-32.
15. Burman, E. (2008). Developments: Child, image, nation. New York, NY, Routledge/Taylor & Francis Group; US.
16. Bush, L. (2014). "In the Best Interest of the Child: Psychological and Ethical Reflections on Traditions, Contexts, and Perspectives in Pediatric Clinical Genomics." American Journal of Bioethics 14(3): 16-18.
17. Cannella GS, Swadener BB. Contemporary public policy influencing children and families: "compassionate" social provision or the regulation of "others"? *International Journal of Educational Policy, Research, and Practice: Reconceptualizing Childhood Studies* 2006; 7(1): 81-93.
18. Cavet, J. and P. Sloper (2004). "The participation of children and young people in decisions about UK service development." Child: Care, Health & Development 30(6): 613-621.
19. Chipungu, S. S. (1991). A value-based policy framework. Child welfare: An Africentric perspective. Piscataway, NJ, Rutgers University Press; US: 290-305.
20. Clayton, E. W., L. B. McCullough and L. G. Biesecker (2014). "Addressing the Ethical Challenges in Genetic Testing and Sequencing of Children." American Journal of Bioethics 14(3): 3-9.
21. Coles E, Cheyne H, Rankin J, Daniel B. Getting It Right for Every Child: A national policy framework to promote children's well-being in Scotland, United Kingdom. *The Milbank Quarterly* 2016; 94(2): 334-365.
22. Convention on the Rights of the Child (CRC), G.A. Res. 44/25 (1989). Available at http://www2.ohchr.org/english/law/crc.htm
23. Cooper, P. and C. Cefai (2009). From the needs of children to the need for children: Contemporary values and their implications for the social and emotional wellbeing of children. Promoting emotional education: Engaging children and young people with social, emotional and behavioural difficulties. London, England, Jessica Kingsley Publishers; England: 171-182.
24. Dauda, C. (2013). "Providential understandings of childhood and public policy: The politics of generation, future adulthood and moral regulation of sexuality in liberal democracies." Sexuality & Culture: An Interdisciplinary Quarterly 17(2): 275-304.
25. de Mendonca, M. H. (2002). "[The challenge of health care provision politics for children and adolescents as part of equitable public policies]." Cadernos de Saude Publica 18 Suppl: 113-120.
26. Doek J. Child well-being: Children's rights perspective. In: Ben-Arieh A, et al. (eds). *Handbook of Child Well-Being: Theories, Methods and Policies in Global Perspective*. Dordrecht: Springer Netherlands, 2014: 187-217
27. Eisenberg, L. (1981). "Social context of child development." Pediatrics 68(5): 705-712.
28. Epps, S. and B. J. Jackson (2000). Emerging ethical perspectives in early childhood. Washington, DC, American Psychological Association; US.
29. ESHG. Genetic testing in asymptomatic minors: Recommendations of the European Society of Human Genetics. *European Journal of Human Genetics* 2009; 17: 720-721.

30. Faden, R. R. and M. Powers (2008). "Health inequities and social justice. The moral foundations of public health." Bundesgesundheitsblatt, Gesundheitsforschung, Gesundheitsschutz 51(2): 151-157.
31. Finn, A. and J. Savulescu (2011). "Is immunisation child protection?" Lancet 378(9790): 465-468
32. Fiorvanti, C. M. and M. R. Brassard (2014). "Advancing child protection through respecting children's rights: A shifting emphasis for school psychology." School Psychology Review 43(4): 349-366.
33. Forrest, C. B. and A. W. Riley (2004). "Childhood origins of adult health: a basis for life-course health policy." Health Affairs 23(5): 155-164.
34. Gallagher, J. J. (1992). "The Role of Values and Facts in Policy Development for Infants and Toddlers with Disabilities and Their Families." Journal of Early Intervention 16(1): 1-10.
35. Goodwin, G. P. and J. F. Landy (2014). "Valuing different human lives." Journal of Experimental Psychology: General 143(2): 778-803.
36. Gormley W (2012). *Voices for Children: Rhetoric and Public Policy*. Brookings Institution Press.
37. Ghinea, N., W. Lipworth, I. Kerridge, M. Little and R. O. Day (2014). "Ethics & evidence in medical debates: the case of recombinant activated factor VII." Hastings Cent Rep 44(2): 38-45.
38. Graham H. 'Prevention and Health: Every Mother's Business', A comment on child health policies in the 1970s. *The Sociological Review* 1980; 28(S1): 160-185.
39. Halfon, N., E. S. Barrett and A. Kuo (2010). Translations from human development to public policy. Formative experiences: The interaction of caregiving, culture, and developmental psychobiology. New York, NY, Cambridge University Press; US: 505-530.
40. Halfon N, Hochstein M. Life course health development: An integrated framework for developing health, policy, and research. *Milbank Quarterly* 2002; 80(3): 433-479.
41. Halfon, N., A. Houtrow, K. Larson and P. W. Newacheck (2012). "The changing landscape of disability in childhood." Future of Children 22(1): 13-42.
42. Hardart, G. E. and W. K. Chung (2014). "Genetic testing of children for diseases that have onset in adulthood: the limits of family interests." Pediatrics 134 Suppl 2: S104-110.
43. Hatch JA. Studying children as a cultural invention: A rationale and a framework. In: Hatch JA (ed.). *Qualitative Research in Early Childhood Settings*. Westport, CT: Praeger, 1995.
44. Hayeems, R. Z., F. A. Miller, Y. Bombard, D. Avard, J. Carroll, B. Wilson, J. Little, P. Chakraborty, J. Bytautas, Y. Giguere, J. Allanson and R. Axler (2015). "Expectations and values about expanded newborn screening: a public engagement study." Health Expectations 18(3): 419-429 411p.
45. Helwig, C. C. and E. Turiel (2002). "Civil liberties, autonomy, and democracy: Children's perspective." International Journal of Law and Psychiatry 25(3): 253-270.
46. Howe RB. Do parents have fundamental rights? *Journal of Canadian Studies* 2001; 36(3): 61–78.
47. Huntington C. Rights myopia in child welfare. *UCLA Law Review* 2006; 53(3): 637–99.
48. Huston AC. Connecting the science of child development to public policy. *Social Policy Report* 19(4), *Society for Research in Child Development*, 2005.
49. Janvier, A., K. L. Bauer and J. D. Lantos (2007). "Are newborns morally different from older children?" Theoretical Medicine & Bioethics 28(5): 413-425.
50. Jenson J, Fraser M. *Social Policy for Children and Families: A Risk and Resilience Perspective*. London: Sage Publications Inc., 2006.
51. Kahn A. From child-saving to child development. In: Kamerman SB, Phipps S, Ben-Arieh A, eds. *From Child Welfare to Child Well-Being: An International Perspective on Knowledge in the Service of Policy-Making*. New York: Springer, 2009: 3-7.
52. Kamerman SB, Phipps S, Ben-Arieh A, eds. *From Child Welfare to Child Well-Being: An International Perspective on Knowledge in the Service of Policy-Making*. New York: Springer, 2009.
53. Keren, R., S. Pati and C. Feudtner (2004). "The generation gap - Differences between children and adults pertinent to economic evaluations of health interventions." Pharmacoeconomics 22(2): 71-81.
54. King M (ed.). *Moral agendas for children's welfare*. London: Routledge, 1999.
55. Klaus, A. *Every Child a Lion: The Origins of Maternal and Infant Health Policy in the United States and France, 1890-1920*. Cornell University Press, 1993.
56. Knijn, T. and W. van Oorschot (2008). "The need for and the societal legitimacy of social investments in children and their families: critical reflections on the Dutch case." Journal of Family Issues 29(11): 1520-1542 1523p.
57. Kolhatkar, G. and C. Berkowitz (2014). "Cultural considerations and child maltreatment. in search of universal principles." Pediatric Clinics of North America 61(5): 1007-1022.
58. Kopelman, L. M. (1997). "The best-interests standard as threshold, ideal, and standard of reasonableness." Journal of Medicine & Philosophy 22(3): 271-289.
59. Kopelman, L. M. (2006). "Children as research subjects: moral disputes, regulatory guidance, and recent court decisions." Mount Sinai Journal of Medicine 73(3): 596-604.
60. Koren, G. (2003). "Healthy children as subjects in pharmaceutical research." Theoretical Medicine & Bioethics 24(2): 149-159.
61. LeVine RA, New RS. Introduction. In: LeVine RA, New RS (eds.). *Anthropology and Child Development. A Cross-cultural Reader*. Malden: Blackwell, 2008 l: 1-7.
62. Lucassen, A., G. Widdershoven and S. Metselaar (2014). "Genetic Testing of Children: The Need for a Family Perspective." American Journal of Bioethics 14(3): 26-28.

63. Lundberg O, Yngwe MA, Stjarne MK, et al (2008). The role of welfare state principles and generosity in social policy programmes for public health: an international comparative study. *Lancet* 2008; 372: 1633-1640.
64. Lynch J (2006). *Age in the Welfare State: The Origins of Social Spending on Pensioners, Workers, and Children*. New York: Cambridge University Press.
65. MacNaughton G, Hughes P, Smith K. Young Children's Rights and Public Policy: Practices and Possibilities for Citizenship in the Early Years. *Children and Society* 2007; 21(6): 458-469.
66. Mass, M. and C. van Nijnatten (2005). "Child protection and the conception of parental responsibility." American Journal of Orthopsychiatry 75(2): 220-229.
67. Mayall B. Towards a sociology of child health. *Sociology of Health & Illness* 1998; 20(3): 269-288.
68. Mayall B. Values and assumptions underpinning policy for children and young people in England. *Children's Geographies* 2006; 4(1): 9-17.
69. McLaughlin, J., S. Woods and R. Traustadottir (2013). "Exploring the ethical and social issues associated with children's participation in health and medical research." Developmental Medicine and Child Neurology 55: 61-62.
70. Meaux, J. B. and P. L. Bell (2001). "Balancing recruitment and protection: Children as research subjects." Issues in Comprehensive Pediatric Nursing 24(4): 241-251.
71. Michalski JH. Values and preferences for the 'best policy mix' for Canadian children. CPRN Study F05, 1999.
72. Millei, Z. and A. Jones (2014). "The Australian early childhood curriculum and a cosmopolitan imaginary." International Journal of Early Childhood 46(1): 63-79.
73. Moss P, Petrie P. *From Children's Services to Children's Spaces: Public Policy, Children and Childhood*. London: RoutledgeFalmer, 2002.
74. Nicholson, R. H. (1994). "What is a child worth?" Hastings Center Report 24(1): 5.
75. Nordberg, A., J. Delva and P. Horner (2014). Adolescent pregnancy in Canada: Multicultural considerations, regional differences, and the legacy of liberalization. International handbook of adolescent pregnancy: Medical, psychosocial, and public health responses. New York, NY, Springer Science + Business Media; US: 205-223.
76. OHCHR. General Comment No. 7 (2005: 01/11/2005). Implementing Child Rights in Early Childhood. Available at http://www.ohchr.org/english/bodies/crc/comments.htm [Accessed 2 November 2016].
77. Onheiber, M. D. (1997). "Toward a reorientation of values and practice in child welfare." Child Psychiatry & Human Development 27(3): 151-164.
78. Pellegrino, E. D. (1995). "Toward a Virtue-Based Normative Ethics for the Health Professions." Kennedy Institute of Ethics Journal 5(3): 253-277.
79. Perera, F. (2014). "Science as an early driver of policy: child labor reform in the early Progressive Era, 1870-1900." American Journal of Public Health 104(10): 1862-1871.
80. Phillips, D. (2006). Child care as risk or protection in the context of welfare reform. From welfare to child care: What happens to young children when mothers exchange welfare for work. Mahwah, NJ, Lawrence Erlbaum Associates Publishers; US: 251-259.
81. Piker, A. (2011). "Balancing liberation and protection: a moderate approach to adolescent health care decision-making." Bioethics 25(4): 202-208.
82. Purcell, M. (2010). "Raising healthy children: Moral and political responsibility for childhood obesity." Journal of Public Health Policy 31(4): 433-446.
83. Raphael D. The health of Canada's children: Public policies and the social determination of children's health. *Paediatrics and Child Health* 2010; 15(3): 143-149.
84. Reading, R., S. Bissell, J. Goldhagen, J. Harwin, J. Masson, S. Moynihan, N. Parton, M. S. Pais, J. Thoburn and E. Webb (2009). "Promotion of children's rights and prevention of child maltreatment." Lancet 373(9660): 332-343.
85. Reich M. The politics of agenda setting in international health: Child versus adult health in developing countries. *Journal of International Development* 1995; 7(3): 489-502.
86. Reynaert, D., M. Bouverne-de Bie and S. Vandevelde (2009). "A review of children's rights literature since the adoption of the United Nations Convention on the Rights of the Child." Childhood: A Global Journal of Child Research 16(4): 518-534.
87. Rhode, D. L. (1993). Adolescent pregnancy and public policy. The politics of pregnancy: Adolescent sexuality and public policy. New Haven, CT, Yale University Press; US: 301-335.
88. Reich M. The politics of agenda setting in international health: Child versus adult health in developing countries. *Journal of International Development* 1995; 7(3): 489-502.
89. Rosenbaum S, Mauery DR, Shin P, Hidalgo J (2005). *National Security and US Child Health Policy: The Origins and Continuing Role of Medicaid and EPSDT*. Policy Brief, Department of Health Policy, Georgetown Washington University.
90. Ross, L. F. (2004). "Children in medical research. Balancing protection and access: Has the pendulum swung too far?" Perspectives in Biology and Medicine 47(4): 519-536.
91. Ross, L. F. (2006). Children in Medical Research: Access versus Protection, Clarendon Press.
92. Ross, L. F. (2008). "Ethical and policy issues in pediatric genetics." American Journal of Medical Genetics. Part C, Seminars in Medical Genetics 148C(1): 1-7.
93. Rowland D, Salganicoff A, Keenan PK. The key to the door: Medicaid's role in improving health care for women and children. *Ann. Rev. Public Health* 1999; 20: 403-426.
94. Rycraft, J. R. (2004). "Review of Moral agendas for children's welfare." Child & Adolescent Social Work Journal 21(3): 303-306.

95. Sanders, R. and S. Mace (2006). "Agency policy and the participation of children and young people in the child protection process." Child Abuse Review 15(2): 89-109 121p.
96. Sardell A. *Insuring Children's Health: Contentious Politics and Public Policy*. Boulder, CO: Lynne Rienner, 2014.
97. Sardell, A. (1990). "Child health policy in the U.S.: the paradox of consensus." Journal of Health Politics, Policy & Law 15(2): 271-304.
98. Sribnick EG. The origins of modern child welfare: Liberalism, interest groups, and the transformation of public policy in the 1970s. *Journal of Policy History* 2011; 23(2): 150-176.
99. Seaberg, J. R. (1990). "Child well-being: A feasible concept?" Social Work 35(3): 267-272.
100. Shah S. Does research with children violate the best interests standard? An empirical and conceptual analysis. *Northwestern Journal of Law and Social Policy* 2013; 8(2): 121-173.
101. Solnit, A. J. (2004). "The Bioethics of Children's Rights." Israel Journal of Psychiatry and Related Sciences 41(1): 4-16.
102. Spranzi, M. and L. Brunet (2014). "Personal identity as a form of freedom." Hastings Cent Rep 44(5): 3-4.
103. Stroik S, Jenson J. What is the best policy mix for Canada's young children? CPRN Study F09, 1999.
104. Sylvester, K. (2001). "Caring for our youngest: public attitudes in the United States." Future of Children 11(1): 52-61
105. Tauer, C. A. (2002). "Central ethical dilemmas in research involving children." Accountability in Research 9(3-4): 127-142.
106. Thevenon O. Family policies in OECD countries: A comparative analysis. *Population and Development Review* 2011; 37(1): 57-87.
107. Ungar, W. (2012). "Health technology assessment in child health." Value in Health 15 (4): A206-A207.
108. Ungar, W. J., L. A. Prosser and H. F. Burnett (2013). "Values and evidence colliding: health technology assessment in child health." Expert Review of Pharmacoeconomics & Outcomes Research 13(4): 417-419.
109. Vandergrift K, Bennett S. Children's rights: A framework for health promotion. *Health care Quarterly* 2012; 15(4): 14-17
110. Waldfogel J. What do children need? *Public Policy Research* 2006; 13(1): 26-34.
111. Walsh, T. (1999). "Changing expectations: the impact of 'child protection' on Irish social work." Child & Family Social Work 4(1): 33-41 39p.
112. Westman JC. Children's rights, parents' prerogatives, and society's obligations. *Child Psychiatry and Human Development* 1999; 29(4): 315–28.
113. White, R. B. (1983). "Moral issues in the allocation of health care resources to special child populations." Theoretical Medicine 4(2): 185-191.
114. Wild, M., C. Silberfeld and B. Nightingale (2015). "More? Great? Childcare? A discourse analysis of two recent social policy documents relating to the care and education of young children in England." International Journal of Early Years Education 23(3): 230-244
115. Williams, A. (1997). "Intergenerational equity: an exploration of the 'fair innings' argument." Health Economics 6(2): 117-132.
116. Wolpert, M. (2007). Developing a policy framework for vulnerable children with mental health needs: Challenges and possibilities. Mental health interventions and services for vulnerable children and young people. London, England, Jessica Kingsley Publishers; England: 22-31.
117. Yarrow, A. L. (2011). "A history of federal child antipoverty and health policy in the United States since 1900." Child Development Perspectives 5(1): 66-72.
118. Yoshikawa, H. and M. Currie (2011). Culture, public policy, and child development. Socioemotional development in cultural context. New York, NY, Guilford Press; US: 53-69.
119. Zawati, M. H., D. Parry and B. M. Knoppers (2014). "The best interests of the child and the return of results in genetic research: international comparative perspectives." Bmc Medical Ethics 15.
120. Zelizer, V. A. (1985). PRICING THE PRICELESS CHILD: THE CHANGING SOCIAL VALUE OF CHILDREN, Basic Books.
121. Zivkovic, T., M. Warin, M. Davies and V. Moore (2010). "In the name of the child: The gendered politics of childhood obesity." Journal of Sociology 46(4): 375-392.
122. Zlotnik Shaul, R. and D. Vitale (2009). "Can we afford it?: ethical consideration of expensive drug treatment for neonates and infants." Clinical Pharmacology & Therapeutics 86(6): 587-589.
123. Zlotnik Shaul, R. and W. J. Ungar (2016). "Maximizing the Benefit and Mitigating the Risks of Moral Hazard." Am J Bioeth 16(7): 44-46.

Supplementary References

---

[1] Ross LR, Saal HM, et al. Technical report: Ethical and policy issues in genetic testing and screening of children. *Genetics in Medicine* 2013; 15(3): 234-245.

[2] Hardart GE, Chung WK. Genetic testing of children for diseases that have onset in adulthood: the limits of family interests. *Pediatrics* 2014; 134(S2): S104-110.

[3] Zawati MH, Parry D, Knoppers BM. The best interests of the child and the return of results in genetic research: international comparative perspectives. *BMC Medical Ethics* 15: 1-13.

[4] Clayton EW, McCullough LB, Biesecker LG, et al. Addressing the ethical challenges in genetic testing and sequencing of children. *American Journal of Bioethics* 2014; 14(3): 3-9.

[5] Axford N. Child well-being through different lenses: Why concept matters. *Child and Family Social Work* 2009; 14: 372-383.

[6] Walsh T. Changing expectations: the impact of 'child protection' on Irish social work. *Child & Family Social Work* 1999; 4(1): 33-41.

[7] Onheiber M. Toward a reorientation of values and practice in child welfare. *Child Psychiatry & Human Development* 1997; 27(3): 151-164.

[8] Sanders R, Mace S. Agency policy and the participation of children and young people in the child protection process. *Child Abuse Review* 2006; 15(2): 89-109.

[9] Yarrow AL. A history of federal child antipoverty and health policy in the United States since 1900. *Child Development Perspectives* 2011; 5(1): 66-72.

[10] Ungar WJ, Prosser LA, Burnett HF. Values and evidence colliding: health technology assessment in child health. *Expert Review of Pharmacoeconomics & Outcomes Research* 2013; 13(4): 417-419.

[11] Baird B. Child politics, feminist analyses. *Australian Feminist Studies* 2008; 23(57): 291-305.

[12] Janvier A, Bauer KL, Lantos JD. Are newborns morally different from older children? *Theoretical Medicine & Bioethics* 2007; 28(5): 413-425.

[13] Millei Z, Jones A. The Australian early childhood curriculum and a cosmopolitan imaginary. *International Journal of Early Childhood* 2014; 46(1): 63-79.

[14] Sylvester K. Caring for our youngest: public attitudes in the United States. *Future of Children* 2001; 11(1): 52-61.

[15] Wild M, Silberfeld C, Nightingale B. More? Great? Childcare? A discourse analysis of two recent social policy documents relating to the care and education of young children in England. *International Journal of Early Years Education* 2015; 23(3): 230-244.

[16] Tauer CA. Central ethical dilemmas in research involving children. *Accountability in Research* 2002; 9(3-4): 127-142.

[17] Ross LF. Ethical and policy issues in pediatric genetics. *American Journal of Medical Genetics Part C Semin Med Genet* 2008; 148C:1–7.

[18] Reynaert D, Bouverne de-Bie M, Vandevelde S. A review of children's rights literature since the adoption of the United Nations Convention on the Rights of the Child. *Childhood: A Global Journal of Child Research* 2009; 16(4): 518-534.

[19] Huntington C. Rights myopia in child welfare. *UCLA Law Review* 2006; 53(3): 637–99.
